# The Modulation of the Symbiont/Host Interaction between *Wolbachia pipientis* and *Aedes fluviatilis* Embryos by Glycogen Metabolism

**DOI:** 10.1371/journal.pone.0098966

**Published:** 2014-06-13

**Authors:** Mariana da Rocha Fernandes, Renato Martins, Evenilton Pessoa Costa, Etiene Casagrande Pacidônio, Leonardo Araujo de Abreu, Itabajara da Silva Vaz, Luciano A. Moreira, Rodrigo Nunes da Fonseca, Carlos Logullo

**Affiliations:** 1 Laboratório de Sanidade Animal, Laboratório de Química e Função de Proteínas e Peptídeos and Unidade de Experimentação Animal - RJ, Universidade Estadual do Norte Fluminense Darcy Ribeiro (UENF), Campos dos Goytacazes, Rio de Janeiro, Brazil; 2 Laboratório de Malária, Centro de Pesquisas René Rachou - Fiocruz, Belo Horizonte, Minas Gerais, Brazil; 3 Laboratório Integrado de Bioquímica Hatisaburo Masuda (LIBHM), Núcleo de Pesquisas Ecológicas e Socioambientais de Macaé (NUPEM), Universidade Federal do Rio de Janeiro (UFRJ/Macaé), Rio de Janeiro, Brazil; 4 Centro de Biotecnologia, Universidade Federal do Rio Grande do Sul (UFRGS), Rio Grande do Sul, Brazil; Virginia Tech, United States of America

## Abstract

*Wolbachia pipientis,* a maternally transmitted bacterium that colonizes arthropods, may affect the general aspects of insect physiology, particularly reproduction. *Wolbachia* is a natural endosymbiont of *Aedes fluviatilis,* whose effects in embryogenesis and reproduction have not been addressed so far. In this context, we investigated the correlation between glucose metabolism and morphological alterations during *A. fluviatilis* embryo development in *Wolbachia*-positive (W+) and *Wolbachia-*negative (W−) mosquito strains. While both strains do not display significant morphological and larval hatching differences, larger differences were observed in hexokinase activity and glycogen contents during early and mid-stages of embryogenesis, respectively. To investigate if glycogen would be required for parasite-host interaction, we reduced Glycogen Synthase Kinase-3 (GSK-3) levels in adult females and their eggs by RNAi. GSK-3 knock-down leads to embryonic lethality, lower levels of glycogen and total protein and *Wolbachia* reduction. Therefore, our results suggest that the relationship between *A. fluviatilis* and *Wolbachia* may be modulated by glycogen metabolism.

## Introduction

The transfection of the bacterium *Wolbachia* into insect hosts has been recently performed in order to elucidate the mechanisms of the host-parasite interaction and potentially identify alternatives for disease control [1], [2], since many studies have reported that *Wolbachia* limits dengue virus transmission in *Aedes* mosquitoes [4], [8], [20]. *Wolbachia* is an obligate intracellular Gram-negative microorganism, maternally inherited in a wide range of arthropods, naturally present in 40% of known insect species [48]. *Wolbachia* is able to play the roles of parasite or of symbiont, with a preference for gonadal tissue cells, and. In this process, it causes cytoplasmic incompatibility and parthenogenesis that affect the reproductive cycle of insects, arachnids, crustaceans, and nematodes [3], [21]. A new strain of *Wolbachia* (wFlu) that causes incomplete but high levels of unidirectional cytoplasmic incompatibility has high rates of transmission from mother to offspring, and no apparent fitness costs, indicating that it may disseminate effectively and rapidly through host populations [6]. *Wolbachia* (wFlu) has been found as natural endosymbiont in a neotropical *Aedes fluviatilis* mosquito (Lutz, 1904;  =  *Georgecraigius fluviatilis*) [4], and apparently does not interfere with the development of this mosquito species [6]. This mosquito, like *A. aegypti*, has been found in domestic and peridomestic areas of Brazil, not causing diseases under natural conditions [5]. Therefore, this vector is an excellent experimental model, replacing *A. aegypti* and *A. albopictus* species in the efforts to understand the host-symbiont interaction and the regulation of mutual metabolism, with the added benefit of abolishing the dengue infection risk [7].

Viral resistance and the mechanisms modulating the symbiosis in mosquitoes infected with *Wolbachia* are poorly understood. However, recent studies have shown that *Wolbachia* affects the host energy metabolism and depends on glycolytic intermediates to obtain energy [9]. Proteomic analyses of *Wolbachia* isolated from nematodes have suggested that amino acids [10] and pyruvate may also be used as energy sources [11]. Therefore, these bacteria appear to depend on carbohydrate intermediates to produce ATP, modulating host metabolism to obtain these molecules. Since *Wolbachia* is maternally inherited, this modulation may start during embryogenesis and proceed throughout life [4].

Previous studies demonstrated that carbohydrate metabolism undergoes dramatic changes during arthropod embryonic development, suggesting a dynamic balance of glycogen synthesis and mobilization to maintain embryo integrity [12], [13]. Glycogen is the predominant carbohydrate reserve in most organisms, including insects [14], [15]. In addition, concerning glycogen metabolism, GSK-3 is involved in a wide range of metabolic processes, including the Wnt (wingless) pathway, cell growth and differentiation, as well as the orientation of the segmental polarity during *Drosophila* embryogenesis [17]. GSK-3 integrates a protein complex responsible for β-catenin phosphorylation, promoting its ubiquitination and subsequent degradation. With the activation of the Wnt pathway, the protein degradation complex dissociates. Thus, β-catenin is no longer phosphorylated, accumulating in the cytoplasm [18]. Therefore, GSK-3 acts not only as a core of glucose metabolism, connecting carbohydrate catabolism pathways, but also during embryonic development, as a member of the Wnt pathway [19]. In the present work, the effects of wFlu infection on *A. fluviatilis* embryogenesis were investigated. We demonstrate that, in the presence of the endosymbiont, the mosquito *A. fluviatilis* is dependent on glycogen metabolism during embryonic development, and that GSK-3 interferes in this process.

## Materials and Methods

### Mosquito maintenance

The *Aedes fluviatilis* colony that was used was originally isolated in 1975 in the vicinity of the René Rachou Institute-Fiocruz, Belo Horizonte, Minas Gerais, Brazil [34], [35]. The colony has since been continuously maintained at FIOCRUZ Minas at 27±1°C and 70±10% relative humidity in a 12∶12 h light:dark cycle. Larvae were reared in clean tap water and fed on fish food (Goldfish Colour, Alcon), and adults were fed *ad libitum* with 10% (w/v) sucrose. For egg production, female mosquitoes were blood-fed on anesthetized mice. To perform oogenesis assays, females were kept at 28°C in a BOD humid chamber with a 12∶12 h light:dark cycle for a period of 24 and 48 h after blood meal.

### Ethics statement

This study was carried out in accordance with the recommendations established by the Sociedade Brasileira de Ciência em Animais de Laboratório (SBCAL). The protocols for blood feeding mosquitoes on mice (license number LW-49/10) were approved by the Comissão de Ética no Uso de Animais (CEUA) Fiocruz (license numbers LW-18/12 and LW-38/12).

### Generation of *A. Fluviatilis* strain cured of *w*Flu infection

The wild type (W+) colony of *A. fluviatilis* was cured of its native *Wolbachia* strain *w*Flu by the mass treatment of adult females and males with tetracycline, as previously described [6]. The adult mosquitoes were continuously exposed *ad libitum* to tetracycline hydrochloride (0.1 mg/mL; Sigma) in a 10% sucrose solution for approximately 14 days during three consecutive generations. One thousand adults were treated in each generation to minimize the effects of random genetic drift and to maintain a colony size equivalent to that of a wild-type (W+) *A. fluviatilis* colony. In each generation, individual females were randomly screened using conventional PCR to detect the presence of *Wolbachia*, as described below. Except for the treatment with tetracycline, the wild-type (W+) and the antibiotic-treated (W−) strains of *A. fluviatilis* were otherwise maintained under the same standard insectary conditions. After the withdrawal of tetracycline from the antibiotic-treated (W−) strain of *A. fluviatilis*, no experiments were performed for two further generations, to allow the reacquisition of any environmental colony associated-microbiota and the recovery from any potential side effects of the antibiotic treatment.

### Synchronous egg-laying

This method was performed as previously described [13], but with an oviposition time of 30 min. Hours after egg laying (HAE) were considered as the age assigned to a sample beginning after the 30-min egg laying period. Eggs were maintained wet at 28 °C until the end of embryogenesis or collected at the indicated HAE. For all biochemical assays, embryo development was interrupted by freezing the samples in liquid nitrogen.

### Total protein content

Twenty milligrams of eggs were homogenized in 1 mL of PBS 20 mM pH 7.4 in the presence of a Protease Inhibitor Cocktail (Sigma) and centrifuged at 11,000 x *g* for 5 min. Total protein content was determined as described by Bradford [36], using bovine serum albumin as the standard. Three samples were analyzed for each experimental point.

### Determination of the glucose content

The glucose content of the eggs (20 mg) was enzymatically quantified by a Glucox kit (Doles). After 30 min of incubation at 37°C, the samples were read at 510 nm in a spectrophotometer (Shimadzu UV-1240), according to Vital *et al*. [13].

### Determination of the glycogen content

The glycogen content of eggs was determined as described elsewhere [12]. Twenty milligrams of *A. fluviatilis* eggs were homogenized in 200 mM sodium acetate, pH 4.8, and the supernatants (five replicates from each sample) were incubated with 1 U/mL of α-amyloglucosidase (Sigma) for 4 h at 4°C. The production of glucose was determined as described above. The control for free glucose was obtained from samples without α-amyloglucosidase. The glycogen content was determined using a standard curve submitted to the same conditions.

### Quantification of glucose 6-phosphate

Twenty milligrams of *A. fluviatilis* eggs were homogenized in 250 µL of buffer, 55 mM Tris-HCl pH 7.5, and centrifuged at 200 x *g* for 10 min. Supernatant aliquots (10 µL, in triplicate) were assayed in 385 µL of 55 mM Tris-HCl pH 7.5 containing 5 mM MgCl_2_, 2 mM β-NAD^+^ and 3 U/mL glucose 6-phosphate dehydrogenase (Sigma). β-NADH production was monitored at 340 nm in a spectrophotometer (Shimadzu UV-1240) at 1-min intervals for a 10-min period using a molar extinction coefficient of 6.22 M^−1^, as described by Worthington [37].

### Hexokinase (HK) activity

Twenty milligrams of eggs were homogenized in 250 µL of buffer containing 20 mM Tris-HCl pH 7.5 and centrifuged at 200 × *g* for 10 min. Then, 385 µL of 20 mM Tris-HCl pH 7.5 containing 6 mM MgCl_2_, 0.5 mM ATP, 0.5 mM β-NAD^+^ and 3 U/mL glucose 6-phosphate dehydrogenase were added (Sigma). The reaction was started with 5 µL of 2 mM glucose. β-NADH production was monitored in a spectrophotometer (Shimadzu UV-1240) at 340 nm using a molar extinction coefficient of 6.22 M^−1^, as described by Galina and Da Silva [38].

### Pyruvate kinase (PK) activity

Samples were prepared as described in the HK activity determination procedure. PK activity was measured in 20 mM Tris-HCl pH 7.5, 5 mM MgCl_2_, 1 mM ADP, 0.4 mM β-NADH, 1 U/mL lactate dehydrogenase (Sigma) and the reaction was started with 1 mM PEP. β-NADH consumption was monitored in a spectrophotometer (Shimadzu UV-1240) at 340 nm using a molar extinction coefficient of 6.22 M^−1^, as described by Worthington [37].

### RNA extraction and cDNA synthesis

Twenty-four hours after the start of oviposition, eggs (20 mg) were used to isolate total RNA in TRIzol Reagent (Invitrogen), according to the manufacturer's instructions. RNA quantity and quality were estimated by spectrophotometry (Shimadzu UV-1240) at 260/280 nm. Two micrograms of total RNA were reverse-transcribed at 37°C using the High-capacity cDNA Reverse Transcription kit (Applied Biosystems) with random primers, according to the manufacturer's recommendations.

### Relative transcription of the *Wolbachia* WSP gene

Relative transcription analysis was performed using the StepOnePlus Real-Time PCR System (Applied Biosystems). Quantification of *Wolbachia* was performed by qRT-PCR using primers for the *wsp* gene [4] and primers for the mosquito rp49 gene [39]. Serial dilutions of cDNA were used for curve calibration. Reaction efficiencies between 85 and 100% were determined from calibration curves for each set of primers in 15-µL reactions. Eggs collected at 0, 6, 12, 24 and 48 HAO of non-treated and silenced females were used for the relative quantification of *Wolbachia.*

### *A. fluviatilis* GSK-3 cloning

Degenerated primers (forward: 5′-GTIGCIATHAARAARGTIYTICARGAY-3′, and reverse: 5′-YTTRWRYTCIRTRTARTTIGGRTTCAT-3′) were designed to amplify the GSK-3 conserved regions, according to the method described by Emily-Fenouil *et al*. [40]. The 506-bp fragment amplified was ligated into the pGEM-T plasmid (Promega) to produce the pGEM-AfGSK-3 construct. The identity of the construct was confirmed by DNA sequencing using the Genetic Analyzer 3500 (Life Technologies). The clones were sequenced in both directions at least 3 times.

### Sequence analysis of *A. fluviatilis* GSK-3

The alignment of partial and full nucleotide sequences was generated using the Clustal W multiple sequences alignment program included in BioEdit version 7.2.0 [41]. The accession numbers for the sequences are as follows: *A. fluviatilis* (KF517095), *H. sapiens* (NP_001139628), *A. aegypti* (ABF18078), *C. quinquefasciatus* (XP_001847997) and *A. gambiae* (XP_003436814). The crystal structure of human GSK-3 (pdb 1I09) was used as model to identify amino acid residues involved in active, ATP binding and substrate sites. Phylogenetic analyses were performed with MEGA5 [42]. Since full sequence of *A. fluviatilis* GSK-3 is not available, we performed two phylogenetic analyses using the complete or partial nucleotide sequences of GSK-3 of various organisms. The tree with complete sequences was constructed without the GSK-3 *A. fluviatilis*. The robustness of the resulting groupings was tested by 500 bootstrap replications. The accession numbers for sequences are as follows: *A. fluviatilis* (KF517095), *A. aegypti* (DQ440045), *Anopheles gambiae* (XM_313732.5), *Drosophila virilis* (XM_002055484.1), *Drosophila mojavensis* (XM_002011771.1), *C. quinquefasciatus* (XM_001847945.1), *Ceratitis capitata* (XM_004520340.1), *Drosophila melanogaster* (M_001042794.3), *Pediculus humanuscorporis* (XM_002427112.1), *Tribolium castaneum* (JN602375.1), *Bombyx mori* (AK406424.1) and *Danaus plexippus* (EF554581.1).

### Gene silencing by RNA interference

To ensure dsRNA specificity and to exclude potential non-specific targets, we used the E-RNAi [43], dsCheck [44] and DEQOR [45] programs to determine the dsRNA sequence. However, because the *A. fluviatilis* gene sequences are not available, we performed the analyses based on equivalent regions of the GSK-3 orthologous genes and genome sequences of other species (*A. aegypti*, *D. melanogaster* and *C. elegans*; Accession numbers DQ440045, X70864, NM_060842, respectively). The GSK-3 dsRNA synthesis was carried out using specific primers (forward: 5′- **TAATACGACTCACTATAGGG**AGTTCGGCTAGTACGCATCC-3′ and reverse: 5′-**TAATACGACTCACTATAGGG**GTGGCGATTAAGAAGGTGCTGC-3′) containing a T7 promoter region (in bold). dsRNA synthesis was performed with T7 RiboMAX Express RNAi System (Promega) using 1 µg of template (pGEM-AfGSK3), according to the manufacturer's recommendations. An unrelated gene from *E. coli* (β-galactosidase) was used as a control. The GSK-3 dsRNA was purified using Invisorb Fragment CleanUp (Invitek), according to the manufacturer's recommendations. The dsRNA quantity and quality were estimated by spectrophotometry (Shimadzu UV-1240) at 260/280 nm. Mosquitoes aged between 0 and 3 days were injected with 1 or 2 µg/µL GSK-3 or β-galactosidase dsRNA solutions using the Nanoject II Auto-Nanoliter Injector (Drummond Scientific Company). Mosquitoes were injected in the thorax with three consecutive injections of 69 nL, with a total concentration of 200 or 400 of double-stranded RNA injected in the mosquito abdomen, respectively. Two days later, mosquitoes were fed blood. Three additional days after oviposition, the mosquitoes were considered to have obtained synchronized eggs 0, 6, 12, 24 and 48 HAO. To confirm silencing, 5 mosquitoes were selected randomly and confirmed by real-time PCR. To confirm gene silencing in the eggs, the females injected with dsRNA were blood fed for two days after injection. Four days after feeding, oviposition was induced on wet filter paper. The females were maintained for 1 h in contact with the filter paper, and then, after 1 h of oviposition, the times were recorded. Zero, 6, 12, 24 and 48-h-old eggs were stored in a freezer at −70°C until RNA extraction.

### Mosquito eggshell clarification

Eggs obtained from synchronous egg laying were fixed and clarified according to Trpiš [46] at 0, 6, 12, 24 and 48 HAO. This technique fixes the embryo while making the eggshell transparent. The eggs were viewed in a bright field stereoscope (Leica M205) under 161X magnification for the evaluation of normal embryos and GSK-3 silenced embryos.

### Determination of the duration of embryogenesis

After oviposition, 150 eggs were incubated for 30 h with distilled water for eclosion. The hatched larvae were counted every 2 h, and embryogenesis was considered to finish when 50% of the larvae hatched.

### Viability of the eggs of silenced females

For oviposition, females were induced to lay their eggs in Petri dishes 4 days after blood feeding. The Petri dish was lined with moistened filter paper. Then, 150 eggs were counted immediately after oviposition (zero hour) and sorted as groups of 50 eggs in different Petri dishes. These eggs were soaked in distilled water, and 24 h after the expected time of the end of embryogenesis the total number of hatched larvae was scored.

### Determination of the width of the abdomen of adult mosquitoes

Three-day-old females were injected with GSK-3 or β-galactosidase dsRNA, and blood-fed 48 h after injection. After 3 days, the abdomen size of these females was analyzed. The measurements were obtained using the Stereo Discovery.V8 (Carl Zeiss). Measurements were performed on the mid-abdomen, as shown in the drawing of the mosquito. Widths of the abdomen from mosquitoes injected with the highest and lowest double-stranded concentrations of dsβ-Gal or dsGSK-3 were determined.

## Results

### Embryonic development of *Aedes fluviatilis* with or without the endosymbiont *Wolbachia*

**Figure 1:**
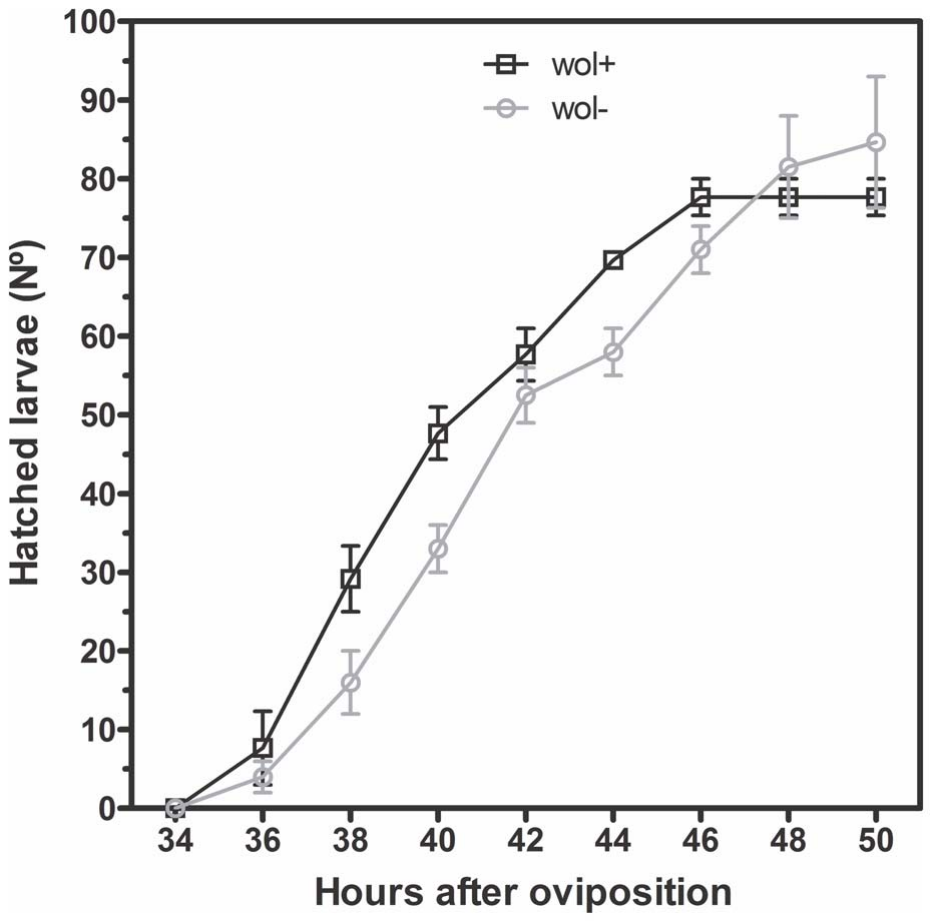
*Wolbachia* does not affect *Aedes fluviatilis* embryo viability. One hundred and fifty eggs were used from each strain and were divided in three dishes with 50 eggs. The hatched larvae were counted at 2-h intervals. The eggs were placed in water, and the number of larvae that emerged was counted. The experiment was carried out at 28°C. The number of eggs that hatched was not significantly different between mosquitoes with *Wolbachia* (Wol^+^/black line) and without *Wolbachia* (Wol^−^/gray line).

**Figure 2:**
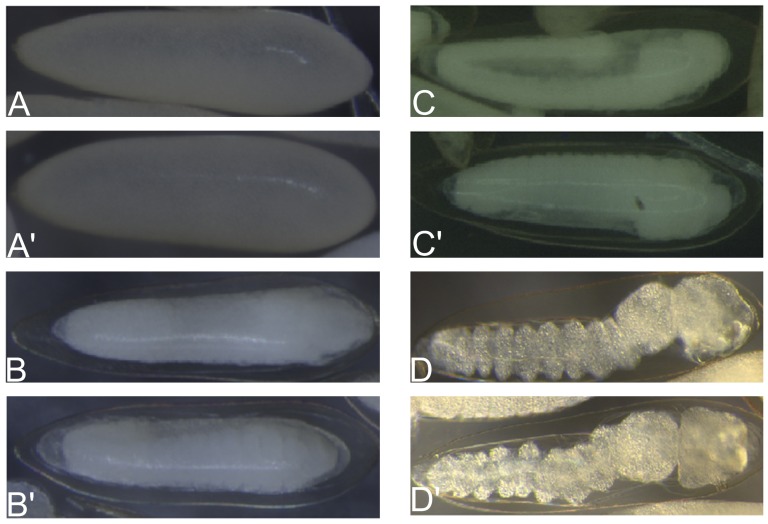
Embryonic development of *Aedes fluviatilis.* Synchronized *A. fluviatilis* eggs were fixed and clarified according to Trpiš [46]. Photos were taken in bright field under 161x magnification. (A) Embryos with 6 HAO; (B) embryos with 12 HAO; (C) embryos with 24 HAO; and (D) embryos with 48 HAO.

To analyze whether the presence of *Wolbachia* affects *A. fluviatilis* embryonic development, the rate of larvae hatching from W+ and W− females was determined. There was no significant difference between the development of W+ and W− embryos incubated at 28°C (Figure 1). The morphological evaluation of 6-h embryos suggests the formation of the syncytial blastoderm stage (Figure 2A). After 12 h, germ band extension and segmentation were observed (Figure 2B). After 24 h, the germ band retraction stage was observed (Figure 2C). Finally, after 48 h, the embryo exhibited several characteristics typical of larvae and was ready to hatch (Figure 2D). Since morphological features and hatching rates appear similar in embryos obtained from W+ and W− females, we investigated the biochemical pathways involved in energy metabolism during embryogenesis.

### Glucose 6-phosphate fate during embryogenesis of *Aedes fluviatilis* in the presence or absence of *Wolbachia*

**Figure 3:**
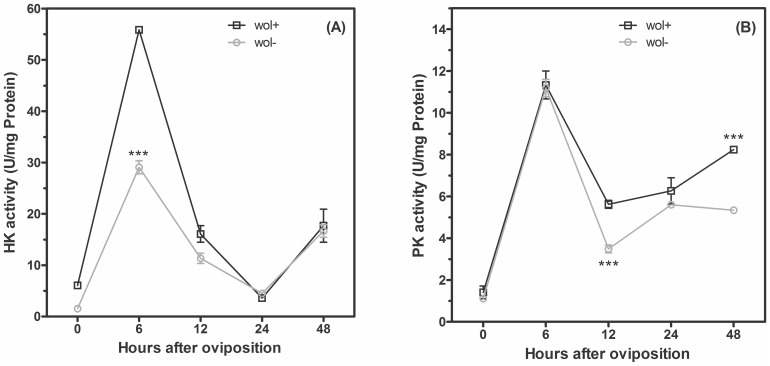
*A. fluviatilis* embryos without *Wolbachia* have reduced hexokinase activity, but demonstrate no difference in pyruvate kinase activity six hours after oviposition. Hexokinase (A) and pyruvate kinase (B) specific activities were measured in egg homogenates at different times after oviposition from mosquitoes with *Wolbachia* (Wol^+^/black line) and without *Wolbachia* (Wol^−^/gray line). Each experiment was replicated three times (*p<0.05; ***p<0.001 ANOVA).

**Figure 4:**
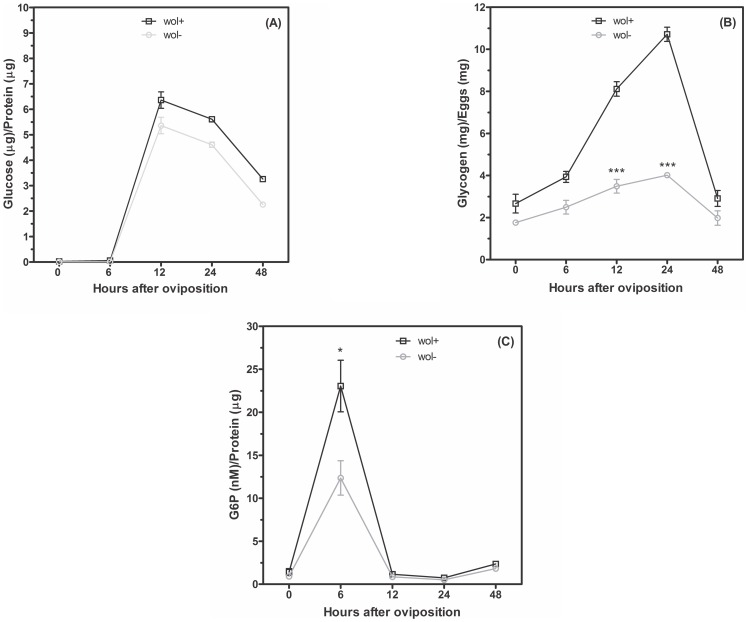
Glucose 6-phosphate formation during embryogenesis is preferential to glycogen formation in *A. fluviatilis* with *Wolbachia*. The glucose (A), glycogen (B) and glucose-6-phosphate (C) concentrations were measured in egg homogenates on different HAO, from mosquitoes with *Wolbachia* (Wol^+^/black line) and without *Wolbachia* (Wol^−^/gray line). Each experiment was replicated three times (*p<0.05; ***p<0.001 ANOVA).

**Figure 5:**
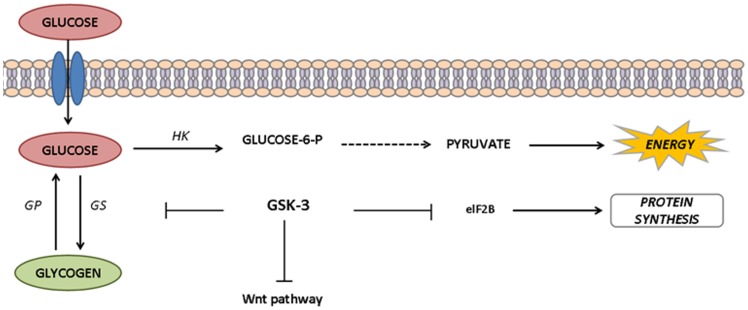
Action of GSK-3 in glucose metabolism. Glycogen synthase kinase-3 is not exclusively involved in glycogen synthase (GS) regulation, but also acts as a downstream component of the Wnt signaling pathway and regulates elF2B, an induction factor for protein synthesis. Glycogen phosphorylase (GP) mobilizes glycogen into glucose subunits. Hexokinase is involved in glucose phosphorylation, acting as an important glycolysis enzyme, the major pathway of pyruvate production. Other possible glucose-6-P routes in carbohydrate metabolism were omitted for simplicity.

The activity of the glycolytic pathway was evaluated determining the activities of two key enzymes in the *A. fluviatilis* embryos. The pattern of pyruvate kinase (PK) activity was correlated with the activity of hexokinase (HK) throughout embryogenesis (Figure 3A, B). The glycolytic flux increased in 6 HAO and decreased after 12 and 24 h, with a slight increase in 48 HAO of development (Figure 3). There was a difference in the activity of HK in 6 HAO compared to W+ and W− embryos (Figure 3A). Within 12 h and 48 h, W+ embryos exhibited higher PK activity compared to W− embryos (Figure 3B). Total glucose content in the embryos exhibited one peak in 12 HAO, which decreased gradually until the end of embryogenesis. There was no significant difference in glucose content between the embryos with and without *Wolbachia* (Figure 4A). The concentration of glucose 6-phosphate (G6P) exhibited the same profile as HK activity. After 6 h, a higher activity of G-6P was observed in W+ embryos, compared to W− embryos (Figure 4C). The overall glycogen profile was as follows: an amount of approximately 2 mg at time 0 following development, gradually increasing until 24 h, at which time the highest peak was observed, and gradually increasing until 48 h, at which time a large drop was observed at this concentration (Figure 4B). W+ embryos exhibited a peak concentration of glycogen at 24 h, which is more than twice the concentration observed in W− embryos, showing that the glucose 6-phosphate in those embryos is intended for glycogen synthesis (Figure 4B). Since glycogen amount display the highest difference between W+ and W− embryos, we cloned and analyzed the function of Glycogen Synthase Kinase-3, a key enzyme involved in multiple roles (Figure 5).

### GSK-3 Cloning and phylogenetic analyses

The GSK-3 proteins contain two highly conserved regions (Emily-Fenouil 1998). Degenerate primers based on these regions amplified a 506-bp product containing the central region of the gene from *Aedes fluviatilis* embryo RNA. The identity of the cDNA sequence was confirmed by comparative sequence analysis using sequence data obtained from GenBank and assigned with accession numbers KF517095.

Multiple sequence alignments derived from partial gene sequences may undermine phylogenetic accuracy, and the decline in this accuracy was associated with the amount of missing data. To confirm the accuracy of the GSK-3 gene phylogenetic trees, we performed two phylogenetic analyses using the nucleotide sequences of either complete or partial GSK-3 genes. One tree was constructed using the neighbor-joining method with partial sequences corresponding to the known region of the *A. fluviatilis* sequence. To confirm the accuracy of phylogenetic inference from partial sequence alignment, a partial GSK-3 *A. fluviatilis* sequence was excluded, and another tree was constructed with the full GSK-3 sequences of other insects. Both methods produced the same branching patterns, with mosquito genes forming a single clade, and both trees had high bootstrap support (data not shown).

### Influence of GSK-3 in *A. fluviatilis* adults with *Wolbachia*

**Figure 6:**
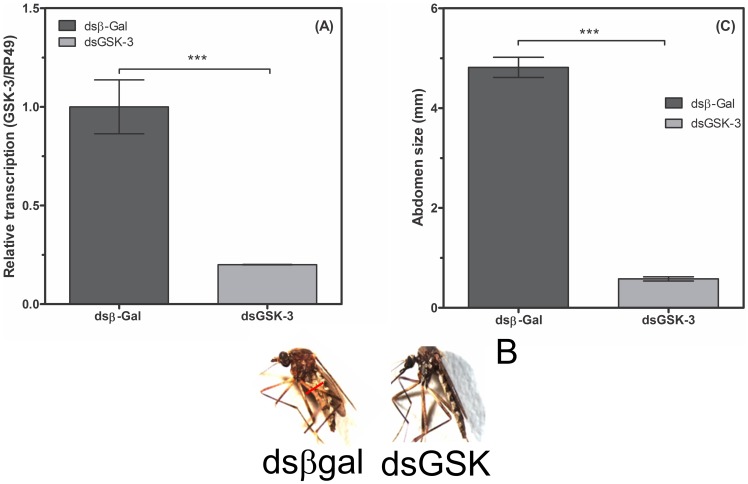
GSK-3 silencing dramatically affects adult *A. fluviatilis* engorgement and development. Unfed Wol+ females were injected with 2 µg of either unrelated double-stranded RNA (β-gal, dashed bar) or double-stranded RNA specific for GSK-3 (black bar) and were fed on blood 24 h after injection. RNA was extracted three days after the blood meal to confirm silencing. After further 3 days, the width of the mosquito's abdomen was measured in both groups under a stereomicroscope. (B) Photos representing the graph are shown in A. (C) Confirmation of silencing by real-time PCR. The results are expressed as the mean ± S.E. of three independent experiments conducted in triplicate (Paired T test, *p* value <0.05 was considered statistically significant).

**Figure 7:**
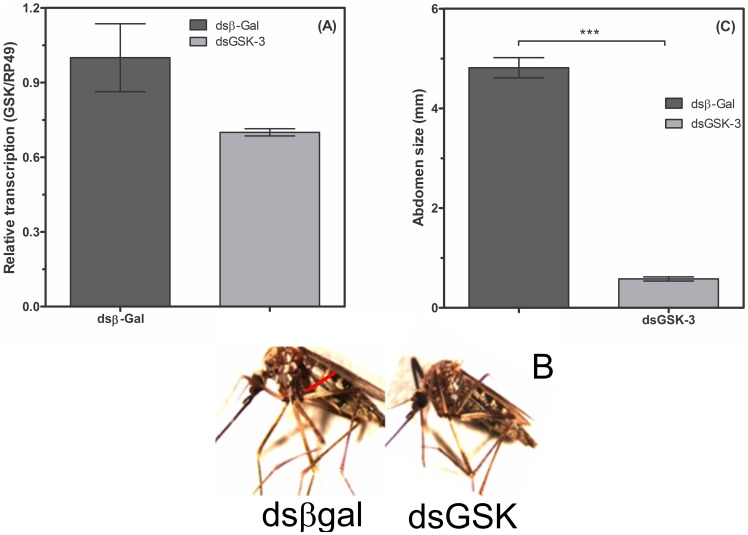
GSK-3 knockdown does not affect adult *A. fluviatilis* engorgement and development. Unfed Wol+ females were injected with 1 µg of either unrelated double-stranded RNA (β-gal, dashed bar) or double-stranded RNA specific for GSK-3 (black bar) and fed on blood 24 h after injection. RNA was extracted three days after the blood meal to confirm silencing. After an additional 3 days, the mosquito abdomen width was measured in both groups under a stereomicroscope. (B) Photos representing the graph are shown in A. (C) The confirmation of silencing by real-time PCR. The results are expressed as the mean ± S.E. of three independent experiments, in triplicates. (Paired T' test, *p* value <0.05 was considered statistically significant).

To evaluate the effect of GSK-3 in glycogen metabolism, the suppression of the enzyme expression was performed through microinjection of 2 µg of dsRNA into 3-day-old females. After blood feeding, mosquitoes silenced for GSK-3 showed diminished abdominal distension, and ovaries did not develop properly; however, the ovaries of females injected with unrelated dsRNA showed normal development. The width of the abdomens in mosquitoes silenced for GSK-3 was reduced by approximately 93% when compared to those injected with unrelated dsRNA (Figure 6B). Additionally, the mosquitoes of the GSK-3 group did not lay eggs (Figure 6B and C). For this reason, it was necessary to reduce the amount of dsRNA to obtain eggs and to evaluate the effect on insect embryogenesis. GSK-3 transcription was reduced by 30%, while oviposition was reduced by 90% using 1 µg of dsRNA (Figure 7A). The abdominal distension measurements of dsβ-Gal or dsGSK-3 dsRNA adults were similar (Figure 7B and C).

The identification of non-specific targets is essential for minimizing off-target effects to better determine gene function by RNAi studies. Considering that the genomic and gene sequences of *A. fluviatilis* are not available, we performed this analysis using the equivalent gene of other species. We used the 506-bp fragment of *A. fluviatilis* GSK-3 to select the equivalent GSK fragments of *A. aegypti*, *C. elegans* and *D. melanogaster*. These fragments were compared against the respective genome with the E-RNAi, dsCheck and DEQOR programs to determine the specificity of the dsRNA used in our experiment. This analysis showed that the probabilities of off-target silencing effects during *A. fluviatilis* GSK-3 gene silencing are very low.

### The role of GSK-3 in *A. fluviatilis* embryos with *Wolbachia* as determined by RNA silencing

**Figure 8:**
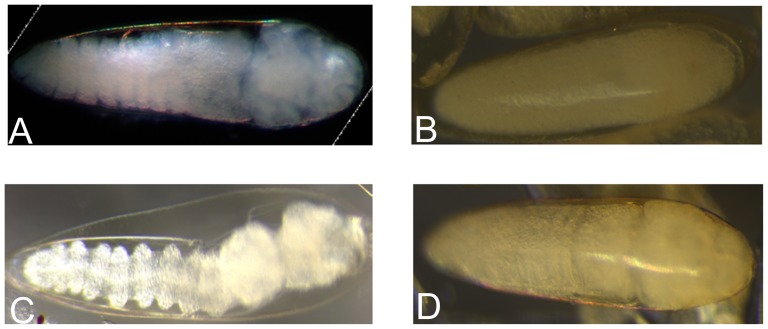
GSK-3 silencing affects *A. fluviatilis* embryo development. Unfed Wol^+^ females were injected with 1 µg of either unrelated double-stranded RNA (β-gal, dashed bar) or double-stranded RNA specific for GSK-3 (black bar) and fed on blood at 24 h after injection. Synchronized *A. fluviatilis* eggs were obtained three days after blood meal. The eggs were fixed and clarified according to Trpiš [46]. (A) Embryos with 24 HAO; (B) embryos with 24 HAO obtained from silenced females; (C) embryos with 48 HAO; and (D) embryos with 48 HAO obtained from silenced females. The silencing validation was performed on eggs with 24 HAO. (Paired T' test, *p* value <0.05 was considered statistically significant).

The phenotype generated by silencing GSK-3 in these embryos was observed at 24 and 48 HAO. Few segmented embryos were observed in GSK-3 RNAi embryos when compared with the control group (Figure 8). At 24 h, normal W+ embryos showed germ band retraction and early segmentation, while at 48 h they already were barrel-shaped larva. The viability of GSK-3 RNAi embryos decreased by 70% compared to non-silenced embryos.

### GSK-3 controls the concentrations of glycogen, protein and *Wolbachia*

**Figure 9:**
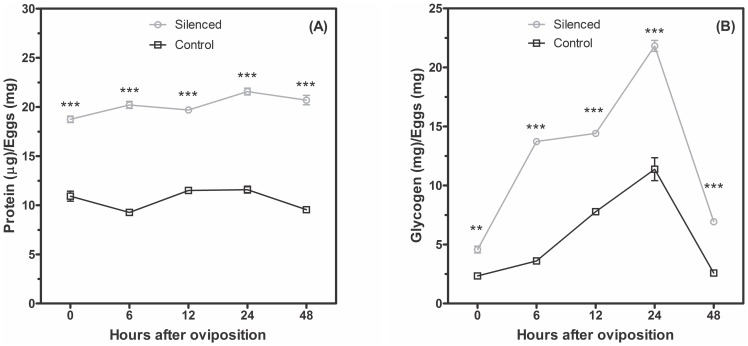
GSK-3 knockdown increases the concentration of protein and glycogen during *A. fluviatilis* embryogenesis. Eggs with 0, 6, 12, 24 and 48 HAO were used to determine the following metabolites. (A) Protein concentration was measured in egg homogenates using the Bradford reagent; glycogen content (B) was enzymatically evaluated by a reaction of α-amyloglucosidase (Glucox kit). The black line represents the normal eggs, and the brown line represents the eggs from GSK-3 silenced mosquitoes. Each experiment was replicated three times (*p<0.05; ***p<0.001 ANOVA).

Quantitative PCR analysis in *A. fluviatilis* eggs confirmed that GSK-3 silencing in adult females resulted in transovarial transmission (data not shown). Eggs were collected at 6, 12, 24 and 48 HAO. Total concentrations of protein, glycogen and wsp relative transcription were analyzed in W+ silenced eggs. The total protein concentration doubled at all times throughout embryogenesis in GSK-3 silenced eggs (Figure 9A).

**Figure 10:**
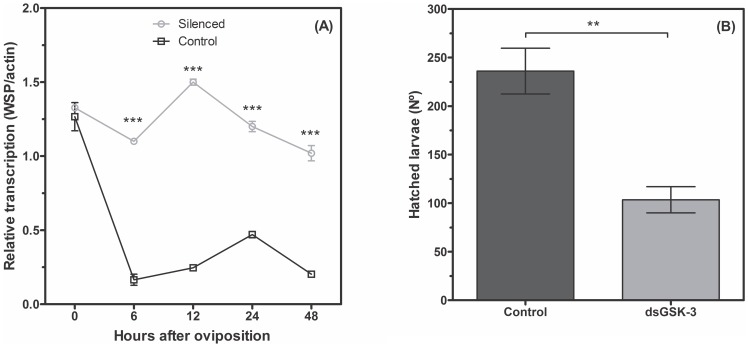
GSK-3 knockdown increases the relative *Wolbachia* concentration and decreases the *A. fluviatilis* embryo viability. (A) Densities of *w*Flu throughout embryogenesis of the *A. fluviatilis* embryos wild-type strain (wolb+). The density of *w*Flu was estimated using real-time quantitative PCR with *Wolbachia*-specific wsp gene primers. Mosquito-specific actin gene primers were used as the reference gene. (B) Three-hundred synchronized eggs were placed on water to evaluate the hatched larvae number. Fifty eggs per plate at 28°C were used. The black line represents the normal eggs and the brown line represents the eggs from GSK-3 silenced mosquitoes. Each experiment was replicated three times. (A) *p<0.05; ***p<0.001 ANOVA. (B) Paired T' test; a *p* value <0.05 was considered statistically significant.

Glycogen distribution in W+ silenced eggs was the same as non-silenced eggs, with the highest peak at 24 h. Similarly to the protein content, glycogen also doubled in GSK-3 silenced eggs, when compared with non-silenced eggs (Figure 9A and B). The most intriguing result was observed in the relative quantification of *Wolbachia*, which showed that the silencing of GSK-3 significantly increased the amount of *Wolbachia* in relation to the non-silenced eggs, except in embryos at zero hour, when silenced and normal eggs displayed the same amount of *Wolbachia* (Figure 10A). *Wolbachia* quantification was performed by relative qRT-PCR since the detection of RNA is more indicative of the presence of viable cells. Considerable effort was made to increase the robustness of our data, including the selection of suitable house-keeping genes (rp49 and wsp) and optimization of reaction parameters used in the qRT-PCR.

## Discussion/Conclusions

*Wolbachia* infects a wide range of organisms, interfering in host embryonic development [47]. However, few studies tried to elucidate the carbohydrate metabolism regulation in this symbiosis. In nematodes, *Wolbachia* provides metabolites as carbohydrates intermediates to the worm, establishing a nutritional mutualism [32].

Glycogen is the major carbohydrate reserve in animals [14] and may be modulated by *Wolbachia* to obtain energy. The glycogen level from W− embryos increased during embryogenesis, while W+ embryos presented an opposite glycogen profile (Figure 4B). Recently, glycogen synthesis was described as mediated by GSK-3 in mosquitoes [13]. Thus, Vital *et al.* [13] reported an accentuated decrease in the GSK-3 activity at the moment of highest glycogen level during *A. aegypti* embryogenesis. Moreover, GSK-3 silencing increased the amount of bacteria in *A. fluviatilis* embryos, compared to the control (Figure 10A). To understand this glycogen accumulation in W+ embryos, GSK-3 knock-down was performed to investigate glycogen metabolism. Initially, silencing was obtained and the females were unable to lay eggs; consequently, ovaries did not develop properly (Figure 6A, B and C).

Injection of a lower amount of dsRNA resulted in a 30% reduction in GSK-3 mRNA levels (Figure 7A), but led to oviposition and larvae hatching, allowing the analysis of adult and embryo phenotypes. Glycogen content increased in *A. fluviatilis* embryos after knockdown (Figure 9B) as well as total protein (Figure 9A), compared to control embryos (Figure 9A and B). Fraga *et al*. [24] observed a similar effect, with an increase in the glycogen concentration after GSK-3 silencing in the beetle *Tribolium castaneum* embryos, when oviposition rate and egg viability were also affected. In *A. fluviatilis*, embryo viability was significantly reduced after GSK-3 silencing (Figure 10B). However, mosquito abdomen size did not differ in GSK-3 silenced mosquitoes, when compared to controls (Figure 7B and C). This result suggests that injection of a lower amount affected only embryogenesis, not ovary development.

GSK-3 is involved in several cellular processes, such as cell cycle, gene transcription and glycogen metabolism control (Figure 5). This enzyme phosphorylates more than 40 substrates, associated to several signaling pathways [25]. One of these pathways is Wingless (Wnt), which modulates embryonic development and energy homeostasis [19], [26], [27]. In GSK-3-silenced *A. fluviatilis*, embryonic developmental was delayed, compared to control embryos (Figure 8C and D). Similarly, Dedeine et al. [22] observed that vitellogenesis was blocked in the wasp *Asobara tabida* without *Wolbachia*, suggesting the involvement of *Wolbachia* in oocyte differentiation, yolk production and germ cell division. GSK-3 silencing also decreased in *Rhipicephalus microplus* oviposition, as well as egg hatching, proposing that enzyme as an essential protein for embryo formation [23].

Syncytial blastoderm was observed within 6 HAO (Figure 2A and A'), and the germ band extension started at 12 HAO (Figure 2B and B'). Around 24 HAO, germ band retraction begins (Figure 2C and C'), similarly to what was observed for *A. aegypti* and *Anopheles albitarsis* [13], [28]. Embryo development is complete within 48 HAO, and larvae are ready to hatch (Figure 2D and D'). Previously, germ band retraction was correlated with increased glycolysis during *A. aegypti* embryogenesis [13]. In the present work, hexokinase and pyruvate kinase activities were measured during *A. fluviatilis* embryogenesis in order to investigate glycolysis. Both enzymes in W− and W+ embryos increased activity after 6 HAO, coinciding with syncytial blastoderm formation (Figure 3A and B). In *A. aegypti* mosquitoes, ILP3 stimulates egg maturation, suggesting that an endogenous ILP stimulates egg maturation by activating the insulin signaling pathway in the ovaries [49].

**Figure 11:**
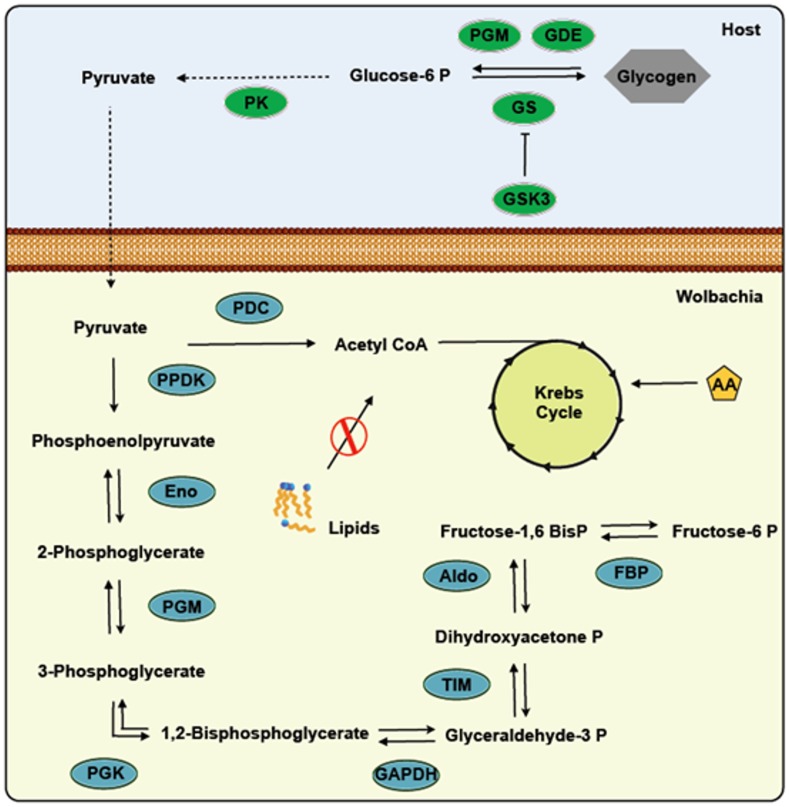
Presumable metabolic pathways retained in *Wolbachia pipientis.* The *Wolbachia* genome was assembled to support the hypothesis of pyruvate internalization from the host. Enzyme annotation is found on methods, a table of which is available at http://tools.neb.com/wolbachia/.

Activation of insulin signaling pathway promotes GSK-3 inhibition by phosphorylation via AKT, contributing to glycogen and protein synthesis [16], [29]. *Wolbachia* interferes in this process, increasing the insulin signaling pathway flux in *Drosophila melanogaster* [30]. The amount of *Wolbachia* is linked to embryo growth and development in *Drosophila simulans*, interacting with the microtubules and cell divisions of these flies [31]. *Wolbachia* may affect glycogen metabolism in *A. fluviatilis* embryos due to a drastic change in glucose metabolism observed in W+ embryos. The enzymes involved in the synthesis and degradation of glycogen are absent in the *Wolbachia* genome, as well as the enzymes of the glycolytic preparatory phase (Table 1). This suggests that *Wolbachia* may be able to internalize host pyruvate, which would be further processed by the pyruvate phosphate dikinase identified in the *Wolbachia* genome. Pyruvate phosphate dikinase uses pyruvate to produce fructose 6-phosphate and acetyl-CoA, as described by Foster *et al*. [32]. Pyruvate is the final product of glycolysis and represents the major substrate of the tricarboxylic acid cycle (TCA) in mitochondria, playing a central role in carbon metabolism regulation. Additionally, pyruvate participates in the catabolic and anabolic pathways, consuming or synthetizing glucose, respectively [11]. The hypothesis of pyruvate internalization by the bacterium is corroborated by the identification of an enzyme linked to lipid degradation in the *Wolbachia* genome, indicating a pyruvate dependence to obtain energy (Figure 11). Despite this finding, the endosymbiont *Wigglesworthia* may oxidize amino acids derived from the host to obtain energy [33]. *Wigglesworthia* is also able to generate carbohydrates through gluconeogenesis, synthesizing glucose from pyruvate [33].

**Table:** **Table 1.** Glucose metabolism correlation between *A. aegypti and A. gambie* mosquitoes and the *Wolbachia* bacterium.

Enzyme/organisms	*Aedes aegypti*	*Anopheles gambiae*	*W. pipientis*
**glycogen branching enzyme**	AAEL010602-PA	AGAP010428-PA	Not
**Glycogen synthase kinase 3**	ABF18078.1	AGAP004443-PB	Not
**Glycogen synthase**	XP_001648704.1	AGAP002586-PA	Not
**Glycogen debranching enzyme**	XP_001660447.1	AGAP001200-PB	Not
**Phosphoglucomutase**	XP_001653384.1	AGAP008305-PA	YP_001975870.1
**Glycogen phosphorylase**	XP_001650265.1	AGAP007939-PA	Not
**Glycogenin**	XP_001649864.1	AGAP007724-PA	Not
**Hexokinase**	XP_001660030.1	AGAP011208-PA	Not
**Glucose-6-phosphatase**	XP_001648840.1	AGAP013732-RA	Not
**glucose-6-phosphate isomerase**	XP_001663180	AGAP012167-PA	Not
**phosphofructokinase**	XP_001652300	AGAP007642-PA	Not

*Wolbachia* surface protein of *Brugia malayi* (wBm0432) is associated with six glycolytic enzymes: fructose-bisphosphate aldolase, triosephosphate isomerase, L-lactate dehydrogenase, enolase, glyceraldehyde-3-phosphate dehydrogenase (G3PD), and phosphoglycerate kinase [9]. In *A. fluviatilis* embryos, glucose 6-phosphate (G6P) concentration follows a similar profile observed for hexokinase and pyruvate kinase activities, showing highest G6P content in W+ embryos, compared to W− embryos (Figure 4C). Total free glucose displayed an opposite profile, compared to G6P content, reaching the highest concentration in late embryogenesis (Figure 4A). *Wolbachia* from *Brugia malayi* displays a defective glycolytic pathway, using gluconeogenesis enzymes to obtain glucose intermediates, like pyruvate [9], [10]. Taken together, our results confirm that the natural symbiotic relation of *w*Flu and *A. fluviatilis* affects the normal host metabolism. This symbiotic relationship may be modulated by glycogen metabolism, which involves GSK-3. Future studies are necessary to understand this mutualism and the *Wolbachia* mechanisms that limits host infection by pathogens.
