# Correction: Modulation of the Symbiont/Host Interaction between *Wolbachia pipientis* and *Aedes fluviatilis* Embryos by Glycogen Metabolism

**DOI:** 10.1371/journal.pone.0109857

**Published:** 2014-09-29

**Authors:** The PLOS ONE Staff

The third sentence in the section titled “Influence of GSK-3 in *A. fluviatilis* adults with *Wolbachia*” of the Results incorrectly refers to Figure 6B instead of 6A. The correct sentence is “The width of the abdomens in mosquitoes silenced for GSK-3 was reduced by approximately 93% when compared to those injected with unrelated dsRNA (Figure 6A).”

The legend for Figure 6 is incorrect. The corrected legend, along with Figure 6, can be viewed below.

**Figure 6:**
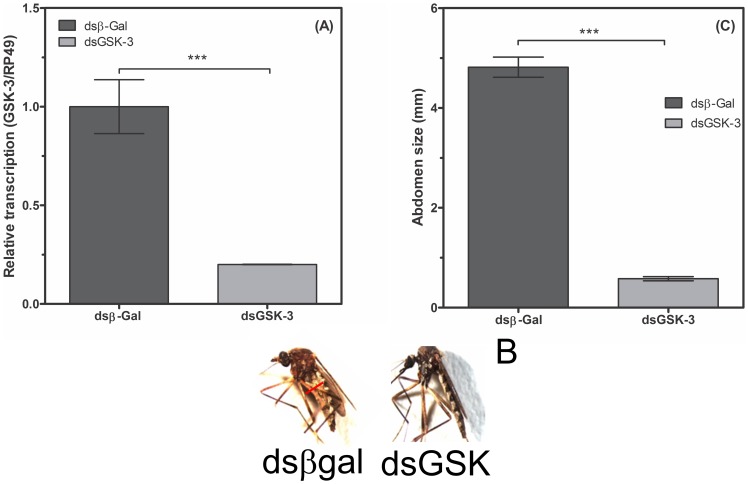
GSK-3 silencing dramatically affects adult *A. fluviatilis* engorgement and development. GSK-3 silencing dramatically affects adult A. fluviatilis engorgement and development. Unfed Wol+ females were injected with 2 mg of either unrelated double-stranded RNA (b-gal, dashed bar) or double-stranded RNA specific for GSK-3 (black bar) and were fed on blood 24 h after injection. RNA was extracted three days after the blood meal to confirm silencing. After further 3 days, the width of the mosquito’s abdomen was measured in both groups under a stereomicroscope. (A) Confirmation of silencing by real-time PCR. (B) Photos representing the graph are shown in C. The results are expressed as the mean 6 S.E. of three independent experiments conducted in triplicate (Paired T test, *p* value, 0.05 was considered statistically significant).

Figure 7C and the legend for Figure 7 are incorrect. The corrected legend and corrected version of Figure 7 can be viewed below.

**Figure 7:**
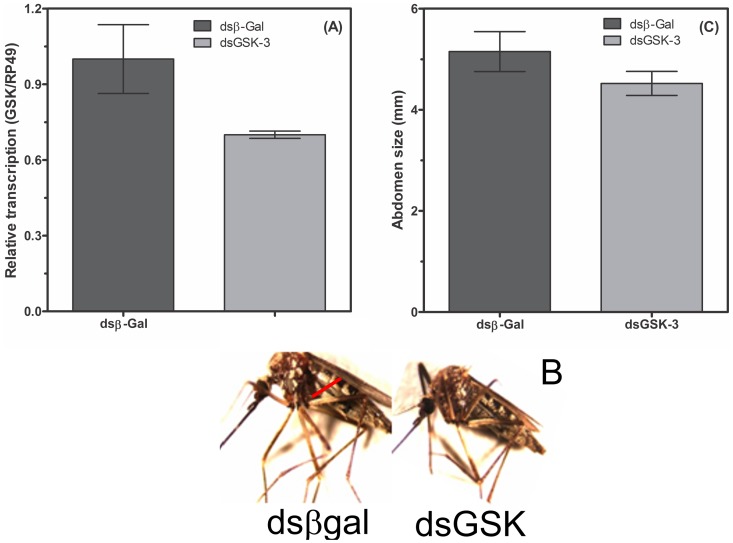
GSK-3 knockdown does not affect adult *A. fluviatilis* engorgement and development. GSK-3 knockdown does not affect adult A. fluviatilis engorgement and development. Unfed Wol+ females were injected with 1 mg of either unrelated double-stranded RNA (b-gal, dashed bar) or double-stranded RNA specific for GSK-3 (black bar) and fed on blood 24 h after injection. RNA was extracted three days after the blood meal to confirm silencing. After an additional 3 days, the mosquito abdomen width was measured in both groups under a stereomicroscope. (A) The confirmation of silencing by real-time PCR. (B) Photos representing the graph are shown in C. The results are expressed as the mean 6 S.E. of three independent experiments, in triplicates. (Paired T’ test, *p* value, 0.05 was considered statistically significant).
